# CMG helicase can use ATPγS to unwind DNA: Implications for the rate-limiting step in the reaction mechanism

**DOI:** 10.1073/pnas.2119580119

**Published:** 2022-01-18

**Authors:** Nina Y. Yao, Dan Zhang, Olga Yurieva, Michael E. O’Donnell

**Affiliations:** ^a^DNA Replication Laboratory, The Rockefeller University, New York, NY 10065;; ^b^HHMI, The Rockefeller University, New York, NY 10065

**Keywords:** CMG helicase, DNA replication, rate-limiting step, staircase model, ATPgammaS

## Abstract

The adenosine triphosphate (ATP) analog ATPγS often greatly slows or prevents enzymatic ATP hydrolysis. The eukaryotic CMG (Cdc45, Mcm2 to 7, GINS) replicative helicase is presumed unable to hydrolyze ATPγS and thus unable to perform DNA unwinding, as documented for certain other helicases. Consequently, ATPγS is often used to “preload” CMG onto forked DNA substrates without unwinding before adding ATP to initiate helicase activity. We find here that CMG does hydrolyze ATPγS and couples it to DNA unwinding. Indeed, the rate of unwinding of a 20- and 30-mer duplex fork of different sequences by CMG is only reduced 1- to 1.5-fold using ATPγS compared with ATP. These findings imply that a conformational change is the rate-limiting step during CMG unwinding, not hydrolysis. Instead of using ATPγS for loading CMG onto DNA, we demonstrate here that nonhydrolyzable adenylyl-imidodiphosphate (AMP-PNP) can be used to preload CMG onto a forked DNA substrate without unwinding.

## Significance

The rate-limiting step of hexameric replicative helicases is ill-understood and this study on the eukaryotic replicative helicase CMG identifies that the catalytic ATP hydrolysis step is not the rate-limiting step, implying that a conformational change may be rate-limiting for DNA-unwinding activity. The results support a “staircase” model of CMG movement along DNA, requiring a large conformational change after ATP hydrolysis. Many studies use ATPγS to “preload” CMG onto DNA. However, this study demonstrates that ATPγS hydrolysis fuels CMG to unwind DNA. To achieve preloading without unwinding, the current study provides an alternative nucleotide analog, AMP-PNP, that is not hydrolyzable and efficiently preloads CMG onto DNA.

The 11-subunit CMG complex (Cdc45, Mcm2 to 7, GINS tetramer) is the replicative helicase of eukaryotic cells (1–3). In common with replicative helicases of all cell types, the motor subunits (Mcm2 to 7) form a hexameric ring that encircles single-stranded (ss)DNA and harnesses adenosine triphosphate (ATP) hydrolysis to track along one strand of ssDNA while excluding the opposite strand, thus acting as a moving wedge to split the parental DNA (4–6). During DNA unwinding, the separated single strands are used as templates by DNA polymerases to duplicate each strand, thereby increasing the DNA content by twofold in preparation for cell division.

Helicases have been categorized into six different superfamilies (SF1 to 6) based on sequence alignments (7). The SF1 and SF2 helicases are monomeric while the SF3 to 6 helicases are hexameric. The SF1 and SF2 helicases work by a “two-step” inchworm mechanism (8, 9), while homohexameric helicases are proposed to function by a staircasing process wherein the hexamer forms a spiral of six subunits encircling the DNA, with sequential hydrolysis of ATP that leads to a hand-over-hand movement of subunits along ssDNA (5, 10–13). Unlike homohexameric helicases, the six (Mcm2 to 7) motor subunits of CMG are each encoded by separate genes having distinct ATP sites. Despite this, recent data indicate that CMG functions by the staircasing mechanism (10).

The current report examines the use of ATPγS by CMG, which is thought to enable CMG loading onto ssDNA but without DNA unwinding (14–18). These expectations are consistent with studies showing that ATPγS is not capable of fueling unwinding by various helicases (19). Indeed, studies have established a 30- to 100-fold lower reactivity of ATP versus ATPγS for enzymatic nucleophilic displacement reactions, attributed to the weak electrophilicity of the thiophosphoryl moiety on the nonbridging thiol of the γ-phosphate (20, 21). However, the results herein reveal the surprising finding that CMG couples ATPγS hydrolysis to DNA unwinding with little effect on rate for 20- to 30-bp forked DNA substrates. The results have important implications for the mechanism of the CMG helicase.

In the cell, replicative hexameric helicases typically require a “loading” factor(s) that helps to place the helicase ring around DNA (5, 12). In vitro, hexameric helicases can self-load onto ssDNA, but are typically very slow to bind DNA and therefore in vitro experiments often use a preincubation step with ATPγS or adenylyl-imidodiphosphate (AMP-PNP) to preload the helicase onto DNA before initiating unwinding with ATP. For example, the *Escherichia coli* DnaB helicase ring can self-load onto DNA by opening/closing upon binding the AMP-PNP nonhydrolyzable analog (22). For CMG, the DNA preloading step is often performed using ATPγS, with the view that this “slowly hydrolyzable” nucleotide analog is not hydrolyzed by CMG for DNA unwinding but can still assist loading of CMG onto ssDNA (14–18).

The assumption that CMG does not hydrolyze ATPγS for unwinding is supported by a history of several examples of helicases that cannot utilize ATPγS. For instance, UvrD, an SF1 helicase, shows no detectable unwinding of DNA with ATPγS (23, 24). Moreover, RecA, also an SF1 helicase, hydrolyzes ATPγS over 1,000-fold more slowly than ATP (19). However, eIF4A, an SF2 monomeric RNA helicase which was long thought to be inactive with ATPγS, was found upon closer inspection to hydrolyze ATPγS at about the same rate as ATP, and was only 10-fold slower in unwinding RNA using ATPγS compared with ATP (25). These findings with eIF4A suggested a rate-limiting conformational change that is slower than the ATP chemical hydrolysis step (25). Furthermore, the study of DNA polymerases with dATPαS, that have a nonbridging thiol on the α-phosphate that is cleaved during DNA polymerization, revealed that the chemical hydrolysis step of DNA polymerization was not rate-limiting for *E. coli* polymerase (Pol) I, suggesting a rate-limiting conformational change in Pol I (20, 26).

We find here that *Saccharomyces cerevisiae* CMG, an SF6 helicase, can indeed hydrolyze ATPγS to fuel the unwinding of DNA. In fact, CMG unwinding of a 20-mer duplex fork and a 30-mer duplex fork using ATPγS is nearly as fast as the use of ATP, implying that the ATP hydrolysis step is not rate-limiting for CMG unwinding. We suggest that a conformational change is rate-limiting and explain this in the context of recent data indicating CMG may function by a staircasing mechanism as proposed in ref. 10, a mechanism in which large protein movements must occur (10, 11, 13). However, we find that the ability of CMG to use ATPγS for unwinding longer duplexes rapidly falls short of comparison with ATP, indicating a crucial step during unwinding of longer duplexes is defective in CMG when using ATPγS.

Whereas CMG can use ATPγS to fuel unwanted DNA unwinding during the helicase preloading step, we demonstrate that use of the nonhydrolyzable ATP analog AMP-PNP enables CMG loading onto DNA without unwinding, suggesting that AMP-PNP may be a better choice than ATPγS for experiments that require preloading CMG onto DNA before unwinding is initiated.

## Results

### CMG Can Unwind Forked DNA Using ATPγS.

**Fig. 1.**
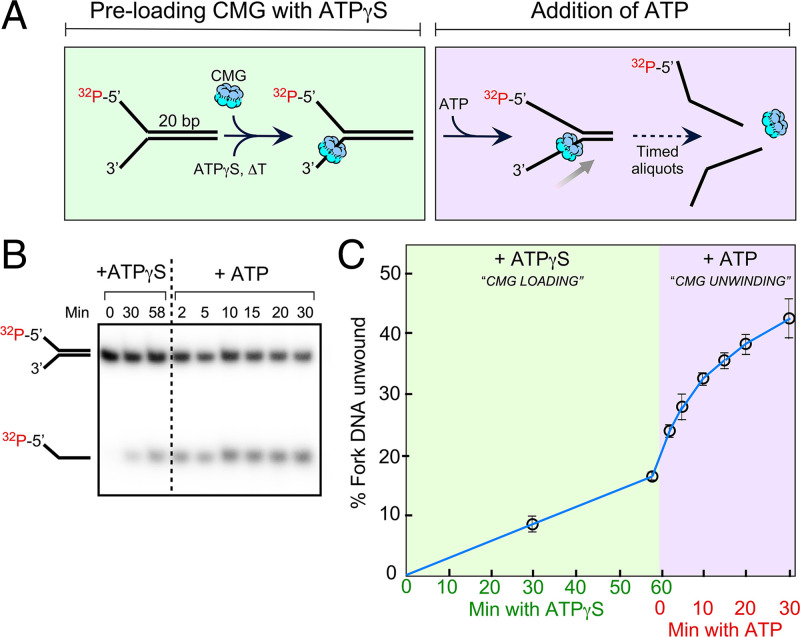
CMG can use ATPγS to unwind a DNA fork. (*A*) Unwinding of a 20-bp forked DNA by CMG. The scheme (*Left*) explains that the forked DNA is preincubated with CMG and 0.1 mM ATPγS for various times, and then 5 mM ATP is added to initiate unwinding (*Right*). (*B*) Native 10% polyacrylamide-gel electrophoresis (PAGE) of the helicase assays. Products formed during the preincubation time with ATPγS are shown in the *Left* three lanes, and reaction times with ATP are shown in the *Right* six lanes. (*C*) Quantitation of the gel data in *B*; error bars show the SEM.

**Fig. 2.**
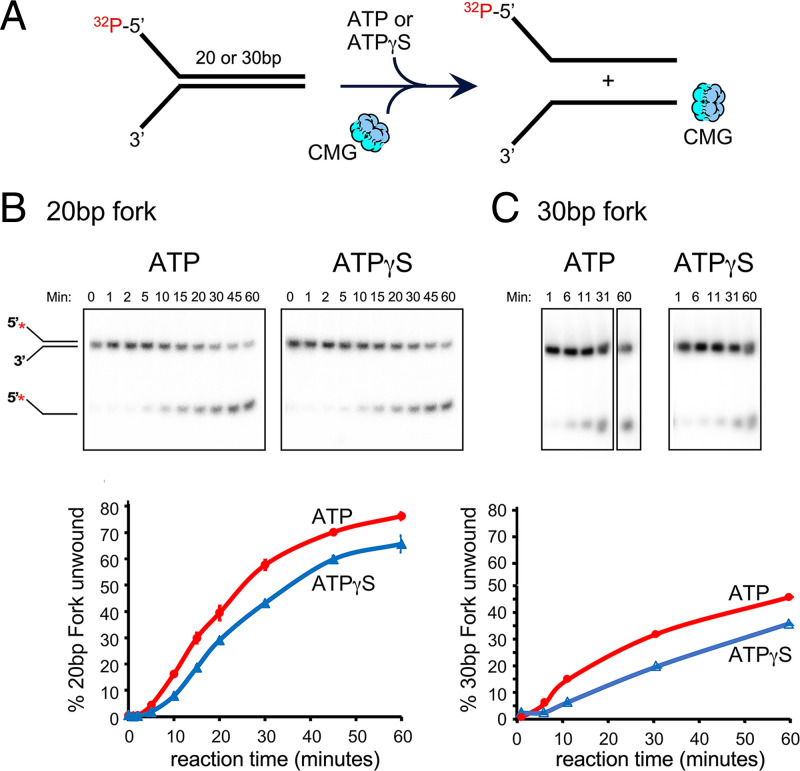
CMG unwinds 20- and 30-bp forked DNAs of different sequences at nearly the same rate using ATPγS or ATP. (*A*) Scheme of the assays. No preloading step was used. Instead, ATP or ATPγS was present and CMG was added directly to assays. (*B*) Native PAGE of CMG helicase assays using either ATP or ATPγS to unwind a 20-bp forked DNA assembled from Y20 leading and lagging oligos (*SI Appendix*, Table S1). (*B*, *Bottom*) Quantitation of the results; assays were performed in triplicate and error bars show the SEM. (*C*) Native PAGE of CMG helicase assays using either ATP or ATPγS, to unwind a 30-bp forked DNA having distinct sequences from the 20-bp fork, and assembled from N30 leading and lagging oligos. (*C*, *Bottom*) Quantitation of the gels is shown. Oligo sequences are in *SI Appendix*, Table S1.

ATPγS is often used to “preload” CMG onto a forked DNA, and this has been presumed to occur without unwinding (10, 14, 15, 17, 18, 27, 28). Recent studies, including those from our laboratory, have shown that maximal CMG preloading onto a 60-bp forked DNA using ATPγS requires over 1 h (14, 15, 18). However, when we performed a similar preincubation using a 20-bp forked DNA, we observed that ATPγS caused CMG to unwind DNA during the 1-h preincubation with ATPγS (Fig. 1; see also Fig. 2). We observed significant unwinding during the preincubation step with ATPγS, prior to adding ATP. This is seen upon gel analysis and quantitation of 30- and 58-min samples taken during preincubation with ATPγS prior to adding ATP (Fig. 1 *B* and *C*). Thus, the extent of unwinding depended on the time of preincubation with ATPγS. This result stands in contrast to the view that CMG cannot use ATPγS to unwind DNA (10, 14, 15, 17, 18, 27, 28). It is important to note that some of the earlier studies that use ATPγS have very short preincubation times, and thus ATPγS-fueled unwinding would not have been of consequence during the preincubation.

To compare the efficiency of CMG unwinding DNA using either ATP or ATPγS, we performed unwinding assays of a 20-bp forked DNA and of a 30-bp forked DNA (Fig. 2). The two forked DNAs are unrelated in their sequence of the duplex region (*SI Appendix*, Table S1). The results show less than a twofold difference in unwinding of the forked DNA using ATPγS versus ATP (Fig. 2). The extent of unwinding is somewhat greater for use of the 20-bp forked DNA than the 30-bp forked DNA, which could be due to the different sequences or lengths.

### The ATPγS Mediated Unwinding by CMG Is Not Due to a Contaminant.

Before proceeding further with the unexpected view that CMG can utilize ATPγS to unwind DNA, we investigated whether a contaminant might explain DNA unwinding with ATPγS. Possible contaminants include 1) the CMG preparation may have a contaminating helicase that can use ATPγS, 2) ATPγS may be contaminated with ATP, and 3) the CMG preparation, DNA preparation, or some buffer component may contain an ATP contaminant.

**Fig. 3.**
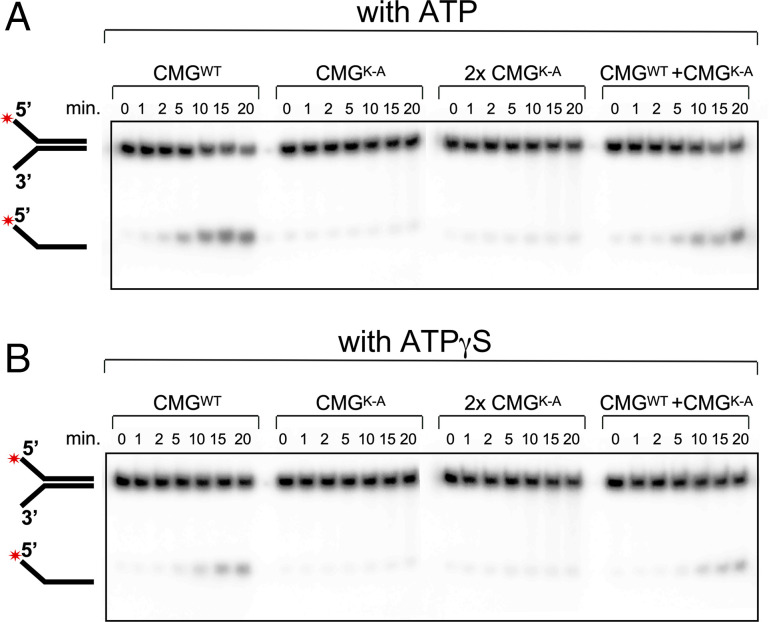
Mutational evidence that unwinding by CMG using ATPγS is not due to a contaminant of ATP. (*A*) Reactions were performed using the N30-bp forked DNA and ATP without preincubation using either wt CMG (first seven lanes) or CMG that carries a mutation in the Walker A box (CMG^K-A^) (second set of lanes). Use of twice the concentration of the CMG^K-A^ mutant is shown in the third set of seven lanes, and use of equal amounts of the CMG^K-A^ mutant plus the wt CMG is shown in the last seven lanes. (*B*) The reactions are the same as explained in *A*, except for use of 0.3 M ATPγS in place of 0.3 M ATP.

First, we made an inactive mutant of CMG in which the Walker A motif active-site lysine in Mcm5 was mutated to alanine (i.e., K-A), and the mutant CMG^K-A^ was purified by the same procedure as wild-type (wt) CMG (*SI Appendix*, Fig. S1). This mutation is reported to nearly completely inactivate *Drosophila* CMG unwinding (2). Indeed, the *S. cerevisiae* CMG^K-A^ mutant does not unwind DNA using ATPγS compared with use of ATP (compare the first seven lanes with the second seven lanes in Fig. 3), even when twice the amount of the CMG^K-A^ mutant was added to the assay compared with the CMG^wt^ control (compare the third seven lanes with the first seven lanes in Fig. 3). Furthermore, addition of the CMG^K-A^ mutant to wt CMG did not prevent ATP- or ATPγS-mediated unwinding by the wt CMG (compare the fourth seven lanes with the first seven lanes in Fig. 3), and therefore the mutant CMG^K-A^ preparation did not contain a general inhibitor of helicase activity. These results rule out a contaminating helicase in CMG preparations that can utilize ATPγS (Fig. 3*B*). The quantitation of the gels of Fig. 3 is shown in *SI Appendix*, Fig. S2.

Second, we note that ATPγS cannot contain contaminating ATP because the synthetic route to form ATPγS cannot yield ATP (29). Nevertheless, we examined three different commercial sources of ATPγS and observed that they each provide the same rate of CMG-mediated DNA unwinding (*SI Appendix*, Fig. S3).

Considering that [^35^S]ATPγS is no longer commercially available, we could not perform equivalent ATP hydrolysis assays. Nor could we use coupled ATPase assays that depend on release of inorganic phosphate (Pi), considering that hydrolysis of ATPγS releases thiophosphate and not Pi. Regardless, the ATPγS must be hydrolyzed because the reaction does not function without either ATP or ATPγS (see below).

Third, we eliminated the possibility that a buffer component of the assay might contain ATP by examining the full reaction in the absence of added ATP. The result, in *SI Appendix*, Fig. S4, shows no detectable unwinding in the absence of added ATP, and therefore none of the components of the assay contained an ATP contaminant.

Finally, we used different CMG preparations that included further column chromatography steps of protein purification, including MonoQ chromatography or MonoQ chromatography followed by a Superose 24 gel filtration column (24 mL prepacked for fast protein liquid chromatography from GE Healthcare), and they showed the same helicase activity with ATPγS as CMG preparations using the two affinity columns.

### Preloading of CMG onto DNA Can Be Achieved with Nonhydrolyzable AMP-PNP.

As mentioned earlier in this report, ATPγS is often used to preload CMG onto a forked DNA (10, 14, 15, 17, 18, 27, 28). Sometimes, the time of preincubation is insufficient for full CMG–DNA binding, and thus would not be expected to yield unwound products. Regardless, we have demonstrated in Figs. 1 and 2 of this report that CMG can use ATPγS for DNA unwinding. Hence, use of ATPγS must be used with caution. Thus, we examined the use of a truly nonhydrolyzable analog, AMP-PNP, to promote CMG loading onto DNA.

**Fig. 4.**
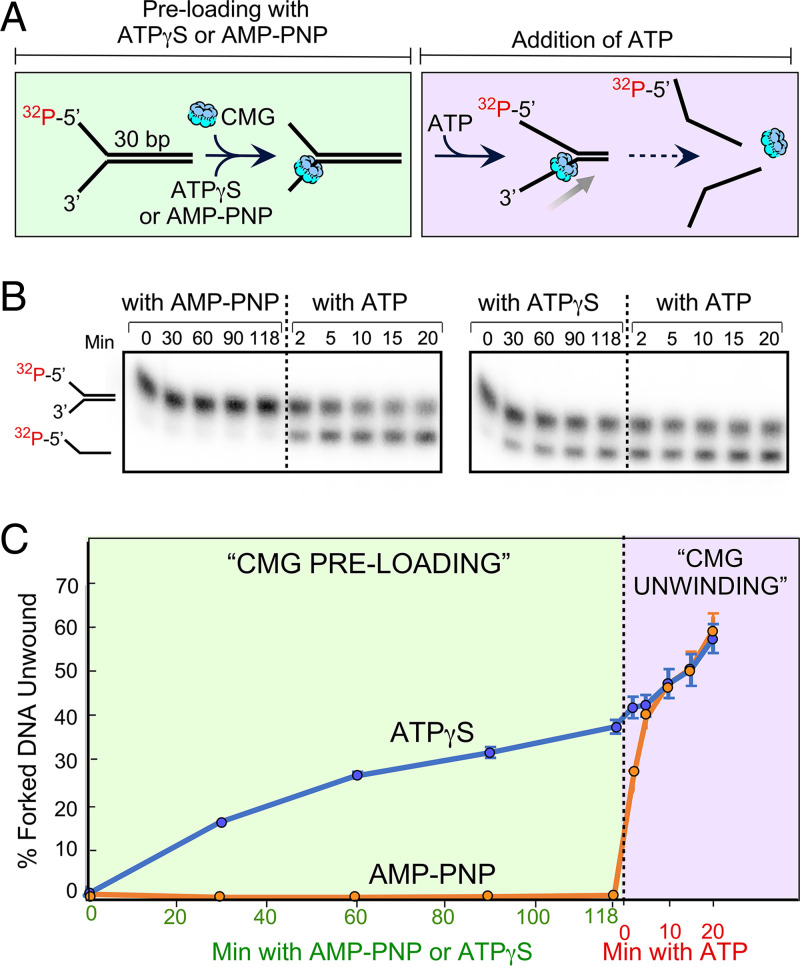
AMP-PNP supports preloading of CMG onto forked DNA. (*A*) Scheme of the assay. Preincubation for CMG loading was with 0.3 mM ATPγS or 0.3 mM AMP-PNP after which 5 mM ATP was added. (*B*) PAGE analysis of products during the preincubation with either ATPγS or AMP-PNP, and after the addition of ATP. (*C*) Quantitation of triplicate assays using ATPγS in which the error bars on the data points represent the SEM.

In Fig. 4, we compared the ability of AMP-PNP and ATPγS to enable CMG preloading onto a 30-bp forked DNA, as signaled by a burst of unwinding upon adding ATP after the preincubation. The results show that substantial DNA unwinding of the 30-bp forked DNA occurred during the preloading reaction containing ATPγS. In contrast, use of AMP-PNP preloads CMG onto forked DNA without unwinding, and provides a burst of unwinding upon adding ATP that equals the extent of unwinding using ATPγS in the preloading reaction prior to addition of ATP.

### Length Dependence of CMG Unwinding Using ATPγS Compared with ATP.

**Fig. 5.**
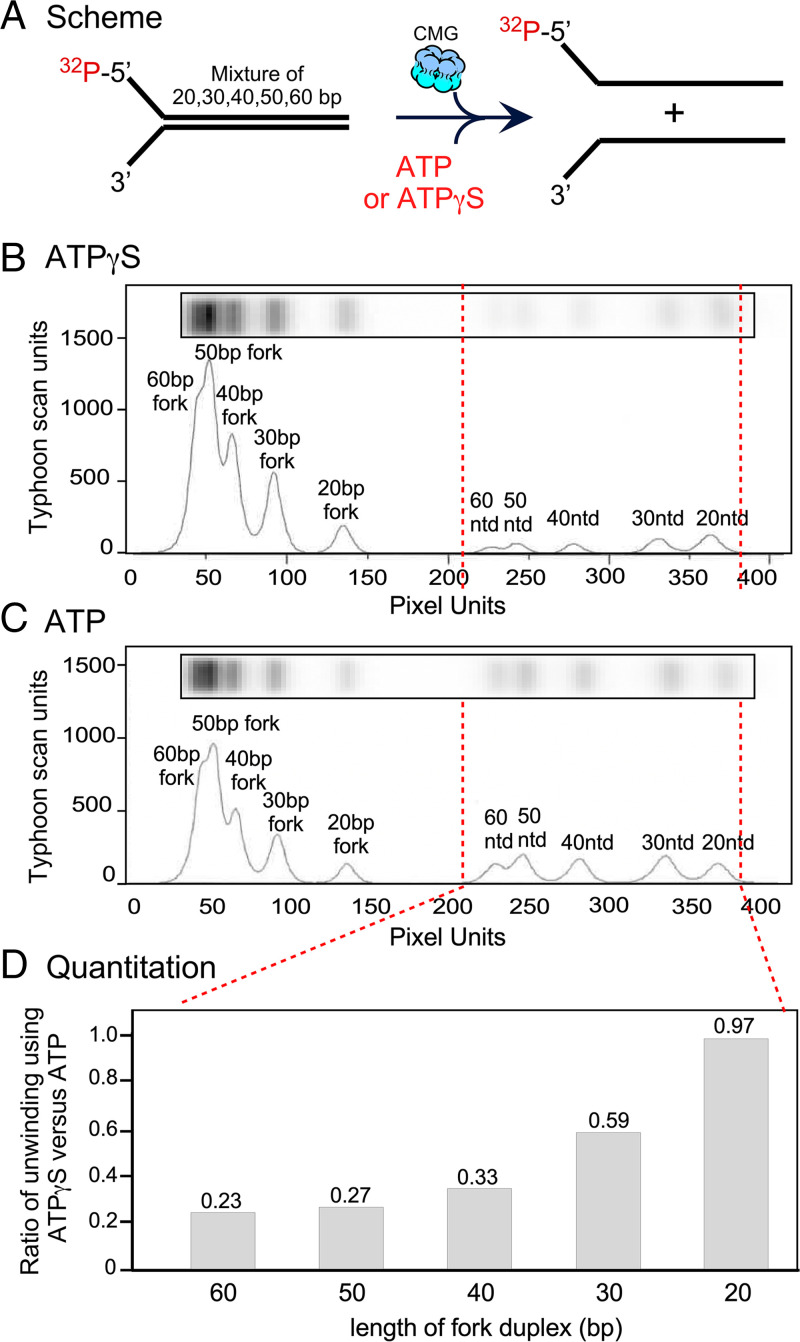
CMG is less able to unwind long products with ATPγS compared with the use of ATP. (*A*) Unwinding reactions were performed using either ATP or ATPγS. (*B*) Substrate and products using ATPγS were analyzed by native PAGE (horizontal slices are shown), and then scanned for intensity by a Typhoon phosphorimager (arbitrary units, but the same units are used for both gels in *B* and *C*). (*C*) The same as *B* except for use of ATP instead of ATPγS. (*D*) Quantitation shows that as the length of the duplex increases, the ratio of DNA that is unwound using ATPγS compared with the unwound DNA using ATP becomes smaller (i.e., ATP results in greater unwinding than ATPγS). The red dashed lines through *B* and *C* point out the unwound DNA peaks. The ratio of the areas of these peaks is presented in *D*.

To examine the relative efficiency of unwinding different lengths of forked duplexes using ATP versus ATPγS, we added CMG to a mixture of forked DNAs having different lengths of the duplex but with the same ssDNA tails, similar to other reports (3, 30, 31). At time points in which most of the substrate is not unwound, the unwound products are assumed to be produced by single-hit kinetics, and thus provides information on the efficiency of the helicase using ATP vs. ATPγS on individual DNA molecules. We added *S. cerevisiae* CMG and ATP or ATPγS to an equal molar mixture of DNA forks having different lengths of the double-stranded DNA stem, either 20, 30, 40, 50, or 60 bp (Fig. 5*A* and Y oligos in *SI Appendix*, Table S1). Each oligo pair was labeled in independent reactions with only slightly different specific activity, which we took into account for quantitation. Comparison of ATP and ATPγS shows much slower unwinding of longer forks compared with the shorter forked DNAs (Fig. 5 *B* and *C*). Notably, while the 20-bp duplex fork is unwound with similar efficiency using ATP or ATPγS, the ratio of unwound DNA using ATPγS to the unwound DNA using ATP becomes smaller as the duplex length is increased (Fig. 5*D*). These results indicate that use of ATPγS has a greater length dependency on unwinding compared with the use of ATP. This could be due to the known slippage of helicases in general (32–34), as has been specifically demonstrated for CMG on ssDNA (14–18), but it is also possible that CMG simply dissociates more frequently from DNA during the use of ATPγS compared with the use of ATP. We presume that the catalytic chemical step, even though not rate-limiting, is still much slower when ATPγS is used versus ATP, and any kind of slippage or DNA loss that could occur during this step will be amplified upon use of ATPγS versus ATP, even though short DNAs do not exhibit this effect.

## Discussion

### Rate-Limiting Step of CMG.

ATPγS is often used to probe whether the ATP hydrolysis step is rate-limiting in an ATP-driven enzyme reaction, as explained in the Introduction. If the chemistry is rate-limiting, ATPγS can slow enzymes 30- to 100-fold or more (20, 21). The results of the current report demonstrate that DNA unwinding by CMG is diminished much less than twofold by ATPγS compared with ATP in unwinding 20- and 30-mer duplex forked DNAs of distinct sequences. This implies that the ATP hydrolysis step is not the major rate-limiting step in the CMG mechanism. We propose that a conformational change is the major rate-limiting step for the CMG helicase. This proposal is consistent with the large motions anticipated for staircasing models of helicase action such as recently proposed for CMG (10). However, we cannot rule out other possibilities, such as product release or association of a third metal ion. It might also be possible that the rate-limiting step in CMG is different for the use of ATP versus ATPγS.

**Fig. 6.**
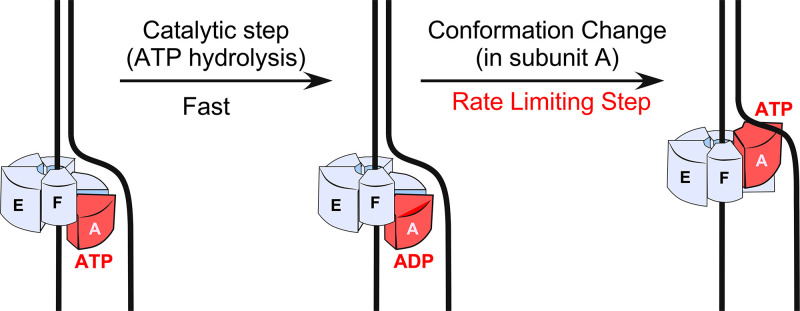
Staircase model of hexameric helicase action. In the staircase model of DNA unwinding, the helicase encircles one strand as a staircase, and the subunit at the bottom of the spiral staircase (i.e., subunit A is shown in red) hydrolyzes ATP and then moves to the top of the spiral and exchanges ADP for ATP to rebind DNA. This process is proposed to repeat around the ring to move the staircase along the ssDNA. Studies of CMG as a staircase indicate unique properties of each ATP site (10) as do studies of individual ATP sites in reconstitution studies (35, 36).

In the staircase model of hexameric helicase action, the ATPase motor domains of each subunit form a spiral around the DNA. ATP hydrolysis causes the ATPase domain at the bottom of the staircase to dissociate from the DNA and move to the top of the staircase (5, 11–13). Exchange of adenosine diphosphate (ADP)/Pi for ATP enables the newly positioned “top subunit” to rebind DNA. Essentially, each subunit acts as an inchworm in sequential fashion within a ring-shaped hexamer. This process is conceptually illustrated for one ATP hydrolytic event in Fig. 6 and, when propagated sequentially around the hexamer spiral, the events propel the ATP-driven hexamer along ssDNA.

It should be noted that CMG is unlike homohexameric helicases in that each MCM subunit of the Mcm2-to-7 motor ring is encoded by a different gene, and thus each ATP site is distinct. Indeed, studies of *Drosophila* CMG indicate that only two Mcm subunits have ATP sites that are absolutely required for helicase action (2). The finding that only two MCM subunits of CMG are required for helicase action suggested possible two-step inchworm models for translocation (37–39), but a recent study makes a strong case for CMG action as a staircasing helicase, with most subunits, but not all, contributing to the helicase staircase process (10).

It should be noted that a spiral staircasing helicase may not need to hydrolyze ATP in a strict sequential order around the ring but could instead be fueled by stochastic ATP firing. In this case, it is even easier to envision that a subset of ATP active subunits might be mainly responsible for CMG translocation. In this regard, a covalently linked homohexamer of the AAA+ ClpX protease that translocates along a peptide chain retains function when one subunit is mutated to be inactive in ATPase activity, although optimal operation may utilize a sequential process (40). Likewise, “salting in” ATPase inactive subunits in an archaeal MCM helicase reduces helicase activity but does not stop the helicase function (41). These findings indicate that the functions of hexameric AAA+ rings are robust and can still operate even when inactive subunits exist within the ring.

### Length Dependence of CMG Unwinding with ATPγS.

We observe that CMG becomes less capable of unwinding a longer duplex (>30 bp) with ATPγS compared with ATP (i.e., Fig. 5), even though CMG uses ATPγS nearly as well as ATP for unwinding 20- and 30-bp duplexes of different sequence compositions. The inability of ATPγS to move CMG over longer distances indicates a processivity deficiency of CMG when using ATPγS.

There are a few obvious explanations for a difference in processivity of CMG using ATP versus ATPγS. First, the intrinsic catalytic rate of ATPγS hydrolysis is slow compared with ATP, but this catalytic chemical step is “kinetically hidden” by the yet slower rate-limiting step (e.g., conformational change). Considering that CMG spends more time in the “hydrolysis state” when using ATPγS compared with ATP, it is possible that there is something about the hydrolysis state that is more conducive to backsliding or DNA dissociation.

All hexameric helicases are well-known to slide backward, or backslide, during unwinding (32–34). In fact, CMG has been demonstrated to backslide (14–18). Therefore, backsliding that is more pronounced using ATPγS versus ATP is a possible explanation of the lower processivity of CMG of >30-bp duplexes when using ATPγS.

Furthermore, it is documented that CMG has an ssDNA gate (42–44), and while the ssDNA gate enabled loading of CMG onto ssDNA with ATP it could conceivably promote dissociation of CMG from DNA using ATPγS. If the ssDNA gate in CMG opens/closes at the step of ATP hydrolysis, the open intermediate state of CMG may be expected to have a longer lifetime when using the slowly hydrolyzable ATPγS compared with ATP, enabling CMG more time to dissociate from DNA using ATPγS.

### AMP-PNP May Be a Better Analog for CMG Loading than ATPγS.

In concluding this report, we show here that AMP-PNP, a nonhydrolyzable ATP analog, can preload CMG onto DNA in the absence of DNA unwinding. The CMG helicase preloading step was the reason that ATPγS was used in past studies, but the current report reveals CMG can, unexpectedly, hydrolyze ATPγS and unwind DNA. Thus, caution is warranted in use of ATPγS for preloading CMG onto DNA, and AMP-PNP may be a safer option for CMG–DNA preloading reactions.

## Methods

### Reagents.

Recombinant yeast CMG was purified from an *S. cerevisiae* expression strain as described (42, 44). Radionucleotides were purchased from PerkinElmer and unlabeled nucleotides were from GE Healthcare. DNA oligonucleotides were purchased from IDT. ATPγS was ordered from Roche for most experiments, but ATPγS was also ordered from Sigma and Tocris.

### Helicase Assays.

Helicase assays were performed using forked DNAs having different duplex lengths and sequences, but they have the same 3′ leading- and 5′ lagging-strand ssDNA arms. The 5′ ssDNA sequence does not bind CMG (17). The oligonucleotides used are detailed in *SI Appendix*, Table S1. Reactions were 10 μL and contained 0.5 nM 5′ ^32^P-labeled forked DNA and 20 nM CMG and were either preincubated with ATPγS (or AMP-PNP) followed by ATP, or were simply initiated using ATPγS or ATP, at the concentrations and times indicated in the figures and legends. Reactions were stopped at the indicated times by adding an equal volume of 40 mM ethylenediaminetetraacetate (EDTA)/1% sodium dodecyl sulfate. Reactions were analyzed on polyacrylamide gels in Tris-borate-EDTA buffer and then gels were exposed to a phosphorimager screen and imaged with a Typhoon FLA 9500 (GE Healthcare). Quantitation was performed using ImageQuant software supplied by the manufacturer (GE Healthcare).

A more detailed description of the methods used in this report is to be found in *SI Appendix*.

## Supplementary Material

Supplementary File

## Data Availability

All study data are included in the article and/or *SI Appendix*.
