# Supplementary material for: CMG helicase can use ATPγS to unwind DNA: Implications for the rate-limiting step in the reaction mechanism
Source: Proc Natl Acad Sci U S A. 2022 Jan 18;119(4):e2119580119. doi: 10.1073/pnas.2119580119 (PMC8794833; doi:10.1073/pnas.2119580119)
Supplement: Supplementary File [file pnas.2119580119.sapp.pdf]

PNAS
www.pnas.org

SI Appendix for
CMG helicase can use ATPγS to unwind DNA; implications for the rate limiting step in the reaction mechanism

By

By Nina Y. Yao[1] , Dan Zhang[1], Olga Yurieva[1,2], and Michael E. O'Donnell[1,2]*

[3] DNA Replication Laboratory, The Rockefeller University, New York, New York, USA
[2] Howard Hughes Medical Institute

[*] Correspondence should be addressed to M.O.D. (odonnel@rockefeller.edu)

This PDF includes:
Table SI
Supplementary Figures 1-4
Supplementary Methods

SI Appendix, Table SI Sequences of oligonucleotides used to make forked DNAs. Oligonucleotide pairs that make the forked duplexes of different sizes used in this report are adjacent in the Table and denoted as Lead or Lag. The black regions form the duplex, poly dT_{40} is the 3' leading ssDNA, shown in red, and lagging ssDNA is shown in green. The asterisks at the 3' nucleotides are phosphorothioate bonds. The N30 pair is a different sequence from the nested set of Y60, Y50, Y40, Y30 and Y20 pairs.

Name	sequence
N30 LEAD	5'GACGCTGCCGAATTCTGGCTTGCTAGGACATTTTTTTTTTTTTTTTTTTTTTTTTTTTTTTTTTTTTTT*T*T*T 3'
N30 LAG	5'GGCAGGCAGGCAGGCAGGCAGGCAGGCAGGCAGGCAGGCTTGTCCTAGCAAGCCAGAATTCGGCAGC*G*T*C 3'
Y60 LEAD	5'CTCGTTTTACAACGTCGTGCTGAGGTGATATCTGCTGAGGCAATGGGAATTCGCCAACCTTTTTTTTTTTTTTTTTTTTTTTTTTTTTTTTTTTTTTT*T*T*T 3'
Y60 LAG	5'GGCAGGCAGGCAGGCAGGCAGGCAGGCAGGCAGGCAGGCAGGTTGGCGAATTCCCATTGCCTCAGCAGATATCACCTCAGCACGACGTTGTAAAAC*G*A*G 3'
Y50 LEAD	5'AACGTCGTGCTGAGGTGATATCTGCTGAGGCAATGGGAATTCGCCAACCTTTTTTTTTTTTTTTTTTTTTTTTTTTTTTTTTTTTTTT*T*T*T 3'
Y50 LAG	5'GGCAGGCAGGCAGGCAGGCAGGCAGGCAGGCAGGCAGGCAGGTTGGCGAATTCCCATTGCCTCAGCAGATATCACCTCAGCACGAC*G*T*T 3'
Y40 LEAD	5'TGAGGTGATATCTGCTGAGGCAATGGGAATTCGCCAACCTTTTTTTTTTTTTTTTTTTTTTTTTTTTTTTTTTTTTTT*T*T*T 3'
Y40 LAG	5'GGCAGGCAGGCAGGCAGGCAGGCAGGCAGGCAGGCAGGCAGGTTGGCGAATTCCCATTGCCTCAGCAGATATCACC*T*C*A 3'
Y30 LEAD	5'TCTGCTGAGGCAATGGGAATTCGCCAACCTTTTTTTTTTTTTTTTTTTTTTTTTTTTTTTTTTTTTTT*T*T*T 3'
Y30 LAG	5'GGCAGGCAGGCAGGCAGGCAGGCAGGCAGGCAGGCAGGCAGGTTGGCGAATTCCCATTGCCTCAGCAGATATCACC*T*C*A 3'
Y20 LEAD	5'CAATGGGAATTCGCCAACCTTTTTTTTTTTTTTTTTTTTTTTTTTTTTTTTTTTTTTT*T*T*T 3'
Y20 LAG	5'GGCAGGCAGGCAGGCAGGCAGGCAGGCAGGCAGGCAGGCAGGTTGGCGAATTCCCA*T*T*G 3'

SI APPENDIX SUPPLEMENTARY FIGURES

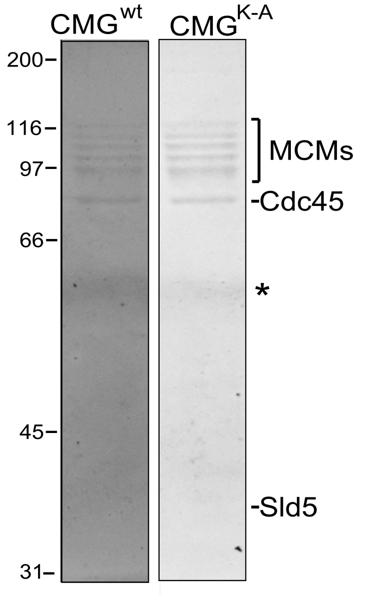

SI Appendix, Figure S1. The active site lysine in the Walker A site GKT motif in Mcm5 was mutated to an Alanine (K-to–A). This CMG$^{K\text{-}A}$ mutation greatly reduces activity of *Drosophila* CMG (1-3), and also highly reduces helicase activity of *S.c.* CMG$^{K\text{-}A}$ (shown in Fig. 3). The wt and mutant CMGs were purified by the same procedures. In the above photograph, the CMG$^{K\text{-}A}$ was analyzed in the same SDS PAGE as the CMG^{wt}, but its concentration was about half that of CMG^{wt}, and therefore brightness was dimmed in order to better visualize Mcm2-7 and Cdc45 subunits of CMG$^{K\text{-}A}$. The asterisk marks the position of a gel artifact that ran as a band across the entire gel, and even between gel lanes.

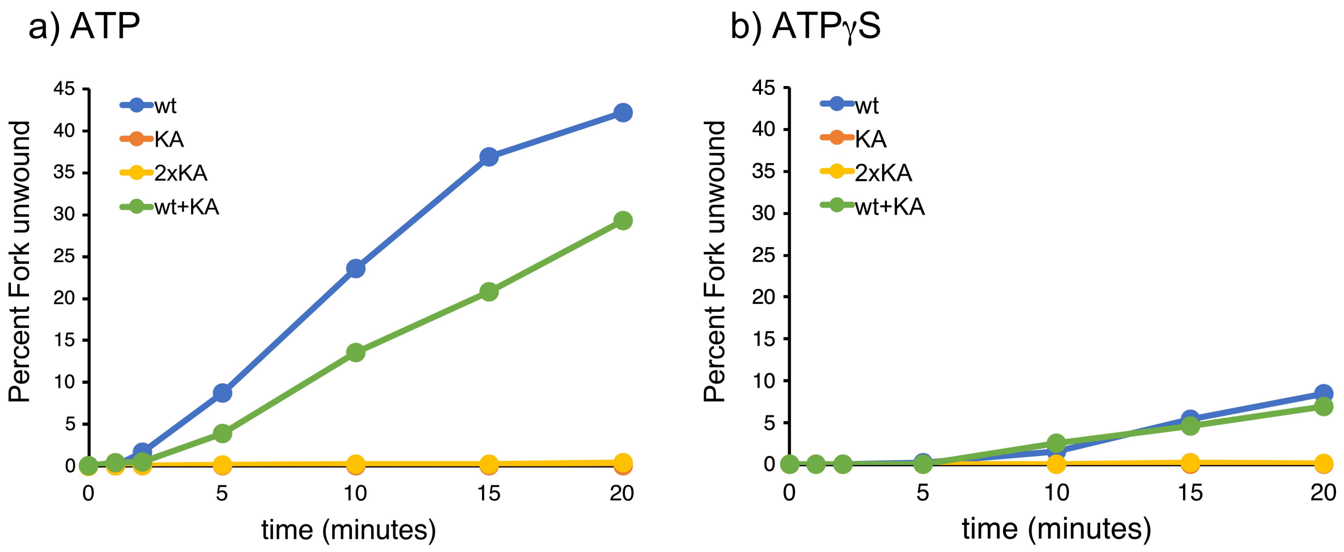

SI Appendix Figure S2. Unwinding activity of a CMG$^{K\text{-}A}$ mutant compared to wt CMG. The plots show the quantitation of the gels bands in the data of **Fig. 3** that compares DNA unwinding of a 30bp fork DNA by a CMG$^{K\text{-}A}$ mutant and wt CMG. **a)** Use of ATP. The CMG$^{K\text{-}A}$ mutant is unable to unwind DNA (orange) even when twice the normal amount is added (yellow). The wt CMG unwinding is shown in blue and the mixture of wt CMG + CMG$^{K\text{-}A}$ mutant is shown in green. **b)** Use of ATPγS. The CMG$^{K\text{-}A}$ mutant is unable to unwind DNA (orange) even when twice the normal amount is added (yellow). The wt CMG unwinding is shown in blue and the mixture of wt CMG + CMG$^{K\text{-}A}$ mutant is shown in green.

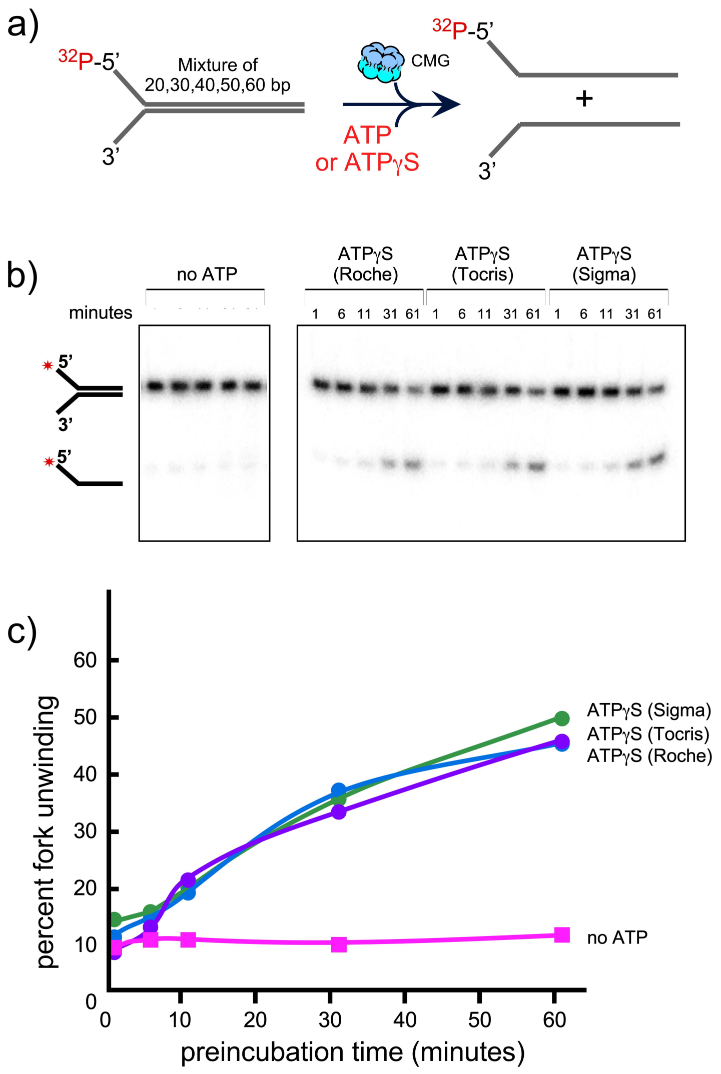

SI Appendix, Figure S3. Use of three different commercial sources of ATPγS as fuel for CMG unwinding of a 30 bp fork that was made by annealing the N30 leading and N30 lagging oligos. 20 nM CMG was added to 0.5 nM DNA as described in Methods and SI Appendix Methods. **a)** Scheme of the assay**. b)** Agarose gel analysis of helicase activity using either ATP, or ATPγS from Roche, Tocris, or Sigma, as indicated above the gel. **c)** Quantitation of the gels.

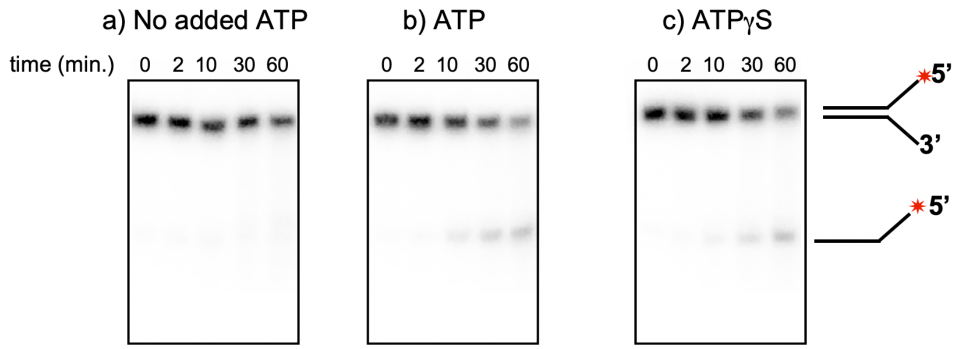

SI Appendix Figure S4. CMG unwinding with ATPγS is not due to contaminating ATP. Reactions were performed in the: **a)** absence of ATP (left panel), with no unwinding observed. Unwinding was observed upon addition of either: **b)** the presence of ATP, or **c)** the presence of ATPγS.

Supplementary Methods

DNAs: DNA oligos were ordered from IDT. The oligos were ordered in pairs, such that when annealed to form a forked DNA, they differ in length and sequence of the duplex portion, but all forked DNAs have the same 3' 40 dT ssDNA leading strand and the same 5' 40 nucleotide lagging strand ssDNA sequence that does not bind CMG (**SI Appendix, Table SI**) (4). This ensures that all CMGs have the same access to all forked DNAs, despite their length of duplex DNA (unless the dsDNA length affects the loading of CMG). The N30 Lead and N30 Lag oligos, when annealed, form a fork that has a distinctive sequence of the 30bp duplex region compared to the Y-series of forks. The Y series of forked DNAs that can be paired to form either 60, 50, 40, 30 or 20 bp of duplex, which are a nested set of sequences in which the 60 bp sequence is that used in (5, 6, 7). The nested set of duplex sequences start at the forked junction, and proceed 20, 30, 40, 50 or 60 bp beyond the junction. Also, all forked DNAs contain three phosphorothioate linkages at the extreme 3' end, to prevent degradation that may occur by a slight contaminant of Pol epsilon, which binds CMG and can be difficult to remove. Typically, Pol epsilon is not observed in a CMG prep that is properly washed with 750 mM KCl on the FLAG column, but even an invisible amount of Pol epsilon can affect a helicase assay that is performed over a long time frame. A Mono Q column removes the last traces of Pol epsilon, and we have used that, where noted, in CMG preps used here.

DNA fork hybridization: First, one strand of the forked DNA was labelled with ^{32}P using polynucleotide kinase according to the manufacturer's instructions. The labeled strand was typically the lagging strand oligo unless mentioned otherwise in the legend. To hybridize the leading and lagging strand oligos, they were mixed in a 1:1.2 molar ratio of unlabeled versus labeled strand, brought to boiling and then slowly cooled in a beaker of water that required about 4 h to reach room temperature. The annealed forked DNA was then gel purified from a native 0.8% PAGE, to ensure that only hybridized DNA was used in the assays. Specific activity of the DNA was determined by counting in a liquid scintillation counter and by measurement of OD in a nanodrop spectrophotometer.

Helicase assays: Two different regimens were used, as illustrated in schemes above the figures and explained in the legends.

Preincubation reactions: For reactions that required preincubation with either ATPγS or AMP-PNP (see figure legends for concentrations), reactions contained 20 mM Tris-acetate, pH 7.6, 5 mM DTT, 0.1 mM EDTA, 10 mM $MgSO_4$, (40 µg/ml BSA, 5% glycerol, 5 mM DTT), 0.5 nM 5'-^{32}P labeled forked DNA substrate (as indicated) and 20 nM CMG, then were preincubated at 30°C in a water bath for the indicated times with 0.1 mM ATPγS or 0.3 mM AMP-PNP. Samples were either removed at indicated times of preincubation, and unwinding was initiated upon adding 5 mM ATP. Reactions were typically scaled such that each timed aliquot would be 10 µl, and one additional reaction would be calculated for the final mixture, to account for any loss of volume during the assay. Reactions were quenched upon adding to an equal volume of 1% SDS, 20 mM EDTA.

Non-preincubation reactions. For reactions that were initiated simply by mixing all components, reaction components were mixed on ice, then brought to 30°C and then initiated upon adding CMG and the indicated deoxyribonucleoside triphosphate. Reactions contained 20 mM Tris-acetate, pH 7.6, 5 mM DTT, 0.1 mM EDTA, 10 mM $MgSO_4$, 40 µg/ml BSA, 5% glycerol, 5 mM DTT, 0.5 nM 5'-^{32}P labeled forked DNA substrate (as indicated), brought to 30°C, and then initiated upon adding the indicated deoxyribonucleoside triphosphate and CMG. Reactions with a mixture of forked DNAs of differerent sizes contained 0.5 nM each DNA fork. Reactions were quenched upon adding to an equal volume of 1% SDS, 20 mM EDTA.

All reactions were analyzed in 10% polyacrylamide gels using TBE buffer and then gels were exposed to a phosphorimager screen and imaged with a Typhoon FLA 9500 (GE Healthcare). Quantitation was performed using the ImageQuant software supplied by the manufacturer (GE Healthcare).

SI references

1. A. Costa *et al.*, The structural basis for MCM2-7 helicase activation by GINS and Cdc45. *Nat Struct Mol Biol* **18**, 471-477 (2011).
2. I. Ilves, T. Petojevic, J. J. Pesavento, M. R. Botchan, Activation of the MCM2-7 helicase by association with Cdc45 and GINS proteins. *Mol Cell* **37**, 247-258 (2010).
3. S. E. Moyer, P. W. Lewis, M. R. Botchan, Isolation of the Cdc45/Mcm2-7/GINS (CMG) complex, a candidate for the eukaryotic DNA replication fork helicase. *Proc Natl Acad Sci U S A* **103**, 10236-10241 (2006).
4. T. Petojevic *et al.*, Cdc45 (cell division cycle protein 45) guards the gate of the Eukaryote Replisome helicase stabilizing leading strand engagement. *Proc Natl Acad Sci U S A* **112**, E249-258 (2015).
5. D. R. Burnham, H. B. Kose, R. B. Hoyle, H. Yardimci, The mechanism of DNA unwinding by the eukaryotic replicative helicase. *Nat Commun* **10**, 2159 (2019).
6. H. B. Kose, N. B. Larsen, J. P. Duxin, H. Yardimci, Dynamics of the Eukaryotic Replicative Helicase at Lagging-Strand Protein Barriers Support the Steric Exclusion Model. *Cell Rep* **26**, 2113-2125 e2116 (2019).
7. Z. Yuan *et al.*, Ctf4 organizes sister replisomes and Pol alpha into a replication factory. *Elife* **8**, e47405 (2019).
